# Inequality in secondary fracture prevention supports prioritisation of fracture liaison services in coastal health plans in England and Wales

**DOI:** 10.1007/s11657-026-01786-7

**Published:** 2026-10-31

**Authors:** L. Pugh, F. Clemeno, R. Ghumman, MK Javaid

**Affiliations:** 1 Sherwood Forest Hospitals NHS Foundation Trust, Nottinghamshire, UK; 2 NDORMS, University of Oxford, Oxford, UK; 3 Falls and Fragility Fracture Audit Programme, Royal College of Physicians, London, UK

**Keywords:** Osteoporosis, Coastal communities, Deprivation, Fracture liaison service, National audit

## Abstract

***Summary*:**

The 2021 UK Chief Medical Officers' annual report highlighted poorer health in coastal communities but did not include osteoporosis or secondary fracture prevention. Using Fracture Liaison Services database (FLS-DB) data, FLSs in coastal areas were shown to have poorer performance and lower parenteral osteoporosis medication recommendation rates, supporting inclusion of FLSs in coastal health improvement plans. A number of studies have highlighted socio-economic deprivation as a predictor of fragility fractures and poorer outcomes after fracture. In parallel, coastal areas are recognised to be areas with poorer health outcomes, in part due to socio-economic deprivation.

**Purpose:**

To describe secondary fracture prevention in terms of FLS provision and quality in coastal vs. non-coastal healthcare settings using routinely collected audit data.

**Methods:**

A cross-sectional, ecological analysis using the missed opportunity counts from the national Fracture Liaison Service (FLS-DB) and Hip Fracture (NHFD) Databases of England and Wales audit data with Office for National Statistics classification of coastal towns.

**Results:**

Results Coastal sites were not significantly more likely to be non-FLS vs FLS providers (38% vs 24%, *p* = 0.18). However, coastal FLSs had higher missed opportunity patient counts than non-coastal FLSs (*p* = 0.02), with lower vertebral identification rates (14.2% vs 20.9%, *p* = 0.02). While treatment rates were similar, coastal FLSs had significantly lower rates of parenteral anti-resorptive therapy recommended (*p* < 0.001).

**Conclusion:**

We have demonstrated a coastal deleterious effect on the quality of secondary fracture prevention of FLS providers as measured by missed opportunities for treatment, vertebral fracture identification and recommendation of parenteral anti-resorptive therapies. Coastal health priority plans in the NHS should prioritise effective FLS provision.

## Background

Osteoporosis and associated fragility fractures can cause significant pain and disability, leading to reduced quality of life for many patients [1]. Major osteoporotic fractures, such as spinal or hip fractures, are also associated with reduced life expectancy. As well as the impact on the patient, the impact on the NHS is substantial, with hip fracture care alone accounting for over £4.3 billion and over 1 million acute hospital bed days annually in the UK. As the population rises, the number of fragility fractures will continue to rise also, with some studies predicting approximately a 30% increase in hip fractures over the course of this decade. In particular, adults aged 50 years and over presenting with a fragility fracture are at high imminent risk of another fracture and this patient group has been prioritised for targeted osteoporosis management [2]. Despite clinically and cost-effective therapies, most adults in this group are not assessed and treated for osteoporosis. Fracture Liaison Services (FLSs) are small healthcare teams that systematically identify, assess, manage and follow up these patients for 12 months in line with local patient pathways [3].

Coastal areas are recognised to age more rapidly than cities, as well as having higher rates of socio-economic deprivation. Both of these characteristics significantly increase osteoporosis and fragility fracture risk [4]. Hence, coastal areas are likely to have a higher need for FLSs than non-coastal areas. The 2021 Chief Medical Officer (CMO) Report on Coastal Health and 2023 CMO Report on Health in an Ageing Society described how coastal areas have a higher burden of physical and mental disease due to in-migration of elderly residents and transient, vulnerable younger population, because of cheaper accommodation, and out-migration of young people due to paucity of local employment options [5, 6]. This is coupled with local recruitment and retention challenges of health and social care staff. In 2023, this has led to the development of the Coastal Health, Livelihood and Environment programme, a partnership of 14 UK governmental departments and agencies, to improve the livelihoods and wellbeing in coastal regions. However, currently the impact of coastal deprivation on health outcomes describes the coastal effect on 20 health care conditions but does not include fragility fractures, and so secondary fracture prevention is not highlighted or included as a coastal health priority. The aim of this study was to describe secondary fracture prevention in terms of FLS provision and quality in coastal and non-coastal healthcare settings using routinely collected audit data.

## Methods

### Data sources

This study was a cross-sectional, ecological analysis of national audit data to explore geographic variation in secondary fracture prevention, specifically examining whether provision of care differs in the UK between coastal and non-coastal providers.

The care gap in secondary fracture prevention was derived using The Royal College of Physicians Fracture Liaison Service Database of England and Wales (FLS-DB) for fractures diagnosed from January to December 2022. The FLS-DB is a mandatory audit collecting patient-level data across 11 key performance indicators [7, 8]. Aggregate data at the FLS level with small number suppression is accessible from the data.gov.uk website. In the NHS, hospitals that provide acute care can be linked to other hospitals as part of a hospital Trust. FLS can cover a single hospital, a Trust, multiple Trusts or a community. We defined each FLS site using the 2022 FLS-DB facilities audit that maps participating FLSs to the healthcare sites they cover. Non-participating Trusts were flagged using the NHFD 2022 dataset. The Office for National Statistics (ONS) dataset, Coastal towns in England and Wales 2020, was used to classify towns as coastal [9]. Any local FLS area that included a coastal town was deemed to be a coastal FLS in this study, regardless of how many non-coastal towns it may also include.

### Outcomes

In 2024, the FLS-DB expanded its reporting to count the number of adults missing treatment annually, ‘missed opportunities’. The missed opportunities calculation is used to quantifying the treatment gap which is well recognised in osteoporosis care following an index fragility fracture. The number of patients is calculated at the hospital/ FLS level as the number of patients who are expected vs. reported on treatment at 1 year post-fracture at each site level annually. NHFD bone treatment data at 120 days is used for sites not participating in the FLS-DB and for the few FLS that do not include hip fractures in their caseload. The missed opportunity expected number of patients on treatment is estimated as five times the number of hip fracture records submitted to the National Hip Fracture Database (Rule of 5) [7], multiplied by 80% identification, 50% treatment recommendation, and 80% adherence 12 months post-fracture, thresholds developed by the FLS-DB clinician and patient advisory group [10]. The missed opportunity reported number of adults on secondary prevention treatment at one year is calculated by multiplying the number of adults identified by an FLS (FLS-DB Key Performance Indicators (KPI) 2 and 3) by the proportion recommended osteoporosis treatment (KPI 7) and proportion who were reported on treatment at the 52-week monitoring visit (KPI 11). To account for the few FLSs that do not routinely include hip fracture patients in their case-mix and sites without an FLS, the 120-day bone treatment rates for hip fractures (NHFD KPI 7) from the sites' NHFD record for the same year were used. Missed opportunities were analysed both as the total counts and as a percentage of the predicted FLS caseload as defined by the Rule of 5. Within the NHS, different types of osteoporosis medication are reimbursed depending on patient characteristics and fracture risk [11]. Osteoporosis medication recommendations were grouped into oral therapies (alendronate, risedronate, ibandronate, raloxifene), parenteral anti-resorptives (denosumab, zoledronate), anabolic (teriparatide, romosozumab), and referred (to a general practitioner or another clinician).

Variable normality was determined using Kolmogorov-Smirnov tests. T-tests and Mann-Whitney U tests were used to compare normal and non-normal variables, respectively. Chi-squared tests were used to compare categorical variables. Analyses were conducted using R and RStudio. *P*-values from multiple comparisons were adjusted using Holm’s method.

## Results

Data from 116 healthcare providers were used in this study, all of which had hip fracture numbers and thus predicted caseloads. Of these, 76 were FLSs and 40 were non-FLS providers (Table 1). Of the 76 FLSs, 68 (89%) submitted data for the 2022 audit. The FLSs that did not submit data for the 2022 audit (and one site with 2022 FLSDB data) also did not have any missed opportunity data available; i.e., 107/116 (93%) had missed opportunity data available.

**Table 1:** NHS sites characteristics by coastal status in 2022

	Coastal Hospital Trusts	Non-Coastal Hospital Trusts	*P* value
All Healthcare providers			
Number of Acute Trusts	33	83	
Number of hip fractures from NHFD per Trust (median (IQR))	458 (362–588)	356 (276–491)	
Missing opportunity data			
Number of Acute Trusts	32	75	
Missed opportunities (counts) per Trust	948 (531–1492)	594 (357–1107)	0.005
Proportion of missed opportunities of expected caseload	32 (26.8–78)	31 (26–75.5)	0.199
Only FLS-DB participating FLSs			
N	17 (51.5%)	51 (61.4%)	
Number of patient records submitted to FLS-DB	22105	52013	
KPI 2 – Non spine fracture identification^1^	55.9 (32.5)	54.7 (33.9)	0.898
KPI 3 – Spine fracture identification	14.2 (5.4–22.1)	20.9 (7.8–55)	0.0186
KPI 4 – Time to FLS assessment < 90 days	68 (20.3–80.3)	76.7 (34.9–87.45)	0.462
KPI 5 – Time to DXA assessment < 90 days	22.4 (12.1–41)	31.2 (12.2–58.4)	0.429
KPI 6 – Falls assessment^2^	81.4 (60.3–91.7)	90.9 (54.2–98.0)	0.959
KPI 7 - Bone treatment recommendation	54.6 (20.6)	55.5 (21.5)	0.877
KPI 8 – initiating strength and balance by 16 weeks	5.5 (0–9.4)	2.1 (0–9.75)	0.521
KPI 9 – monitoring within 16 weeks	14.7 (2.4–34.3)	14.9 (0.25–41.8)	0.382
KPI 10 – Initiated treatment within 16 weeks	18.9 (9.4–45.1)	29.5 (1.4–48.6)	0.941
KPI 11 – On treatment at 52 weeks	21.9 (0.2–44.2)	12.4 (0–38.9)	0.828
FLSs with treatment data available	17	48	
Number and proportion of FLS-identified patients recommended different types of osteoporosis treatment:			<0.001
Oral therapies^3^	4502 (20.4%)	9588 (18.5%)	<0.001
Parenteral anti-resorptive^4^	1877 (8.5%)	7270 (14.0%)	<0.001
Anabolic therapy^5^	34 (0.2%)	32 (0.1%)	<0.001
Referred to GP	2627 (11.9%)	8402 (16.2%)	<0.001
Unspecified recommendation	1603 (7.3%)	2860 (5.5%)	<0.001

^1^ KPI 2 relates to non-spine fracture identification that includes low trauma fractures resulting in fractures of any non-spine bone excluding the face, skull, digits and scaphoid, atypical femoral fractures and peri-prosthetic fractures

^2^No minimum dataset is defined for an FLS falls assessment but FLSs are expected to follow local, regional or national guidance

^3^Oral therapies (alendronate, risedronate, ibandronate, raloxifene)

^4^parenteral anti-resorptives (denosumab, zoledronate)

^5^anabolic (teriparatide, romosozumab), referred (to general practitioner to another clinician). The *p*-values obtained for each treatment recommendation were adjusted using Holm’s method to account for multiple comparisons. Data are expressed as N (%)

**Fig. 1:**
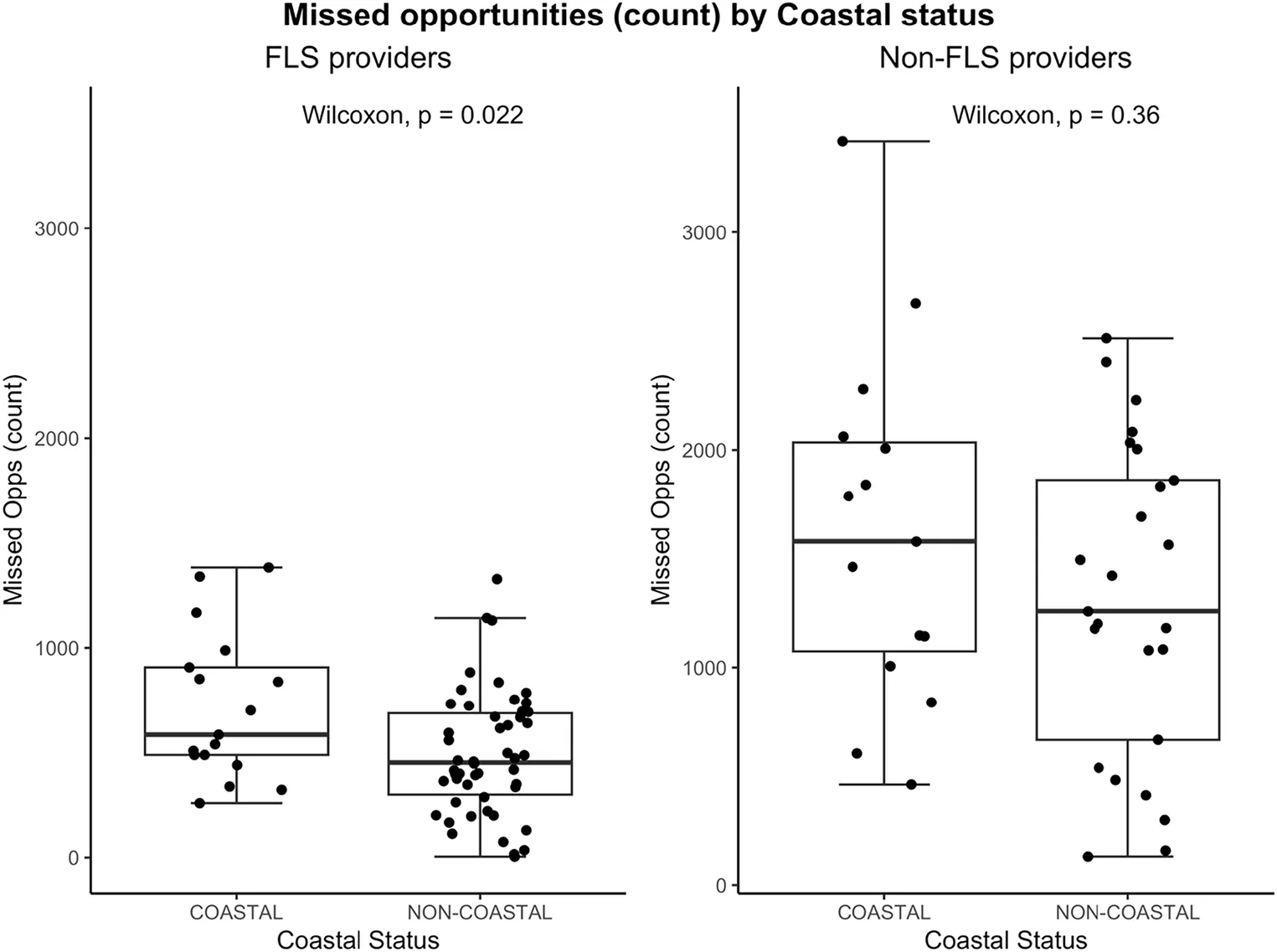
Total number of missed opportunities, by FLS participation and coastal status

Of the FLS and non-FLS sites included in this study, 24% and 38% were classified as coastal (*p*-value = 0.18), so coastal sites are not significantly more likely to be non-FLS providers. However, coastal sites had significantly higher missed opportunities than non-coastal sites. This difference was only significant in FLS providers (*p* = 0.02), but not in non-FLS providers (*p* = 0.36) (Fig. 1). When comparing the individual FLS-DB KPIs in coastal and non-coastal FLSs, only vertebral fracture identification (KPI-3) differed significantly (14.2% vs 20.9%, respectively, *p* = 0.02). While there was no difference in the rates of treatment recommended (KPI-7) (*p* = 0.88), coastal FLS had significantly different patterns of treatment, including a 40% lower rate of parenteral anti-resorptive therapy (*p* < 0.001) (Table 1). Anabolic prescribing rates were low in both coastal and non-coastal FLS ( < 0.2%).

## Discussion

Using data from national audits, we have demonstrated inequality of effective FLS provision in coastal communities and recommend secondary fracture prevention should be prioritised in UK coastal health plans.

The observed differences in missed opportunities comparing coastal with non-coastal communities exceed that expected from the differences in local hip fracture counts. The care gap extends both to fewer adults receiving secondary prevention and also a mismatch in the type of osteoporosis medication received. Given how common fragility fractures are and their impact on patients, healthcare systems and wider society [1], these findings support that secondary fracture prevention should also be a core disease when measuring coastal health inequality and routinely included in coastal health plans.

Health in an ageing society is focused on improving quality of life rather than longevity [5]. While fragility fractures reduce survival, those who survive live with significantly worse quality of life that is permanent for both hip fractures and fractures at other sites [1]. Hence, improving secondary fracture prevention is a global priority [12] and supported by the recent tier 2 recommendation from the World Health Organisation [13]. The aim of FLSs is systematic delivery of secondary fracture prevention for adults with a recently diagnosed fragility fracture [14]. FLSs have been shown to be both clinically [15] and cost-effective [16, 17]. Despite national and international recommendations for FLSs, a significant gap in FLS provision remains in the UK [18] and internationally [12]. Previous studies have demonstrated lower rates of DXA attendance in those most deprived patients identified by FLSs [19, 20]. Of the differences in FLS KPI, there was a lower proportion of expected patients with vertebral fractures identified by FLSs in coastal communities. As the method of identification is not recorded in the FLS-DB, it is not possible to compare differences in vertebral fracture case-finding methods. The implementation of recent NICE guidance have recommended consideration of systematic vertebral fracture assessment in women aged ≥60 years and men aged ≥70 years [21], as well as AI-enabled vertebral fracture identification from routine CT scans [22] represents an opportunity to enhance vertebral fractures.

The study findings have wider international relevance by highlighting the need for decision makers to specifically target support for sustainable, effective FLS provision in sub-populations with greater health inequality. While this study focused on within-country variation, the findings add to reviews the secondary fracture care gap in low and middle-income countries [23]. This study has used coastal status as a marker for the co-existence of factors such as older population, service inaccessibility and poor workforce availability; in other healthcare systems characteristics such as rural vs urban settings identify distinct sub-populations of inequality unrelated to their proximity to the coast. Finally, translating these findings to prioritisation of FLSs as part of coastal healthcare plans requires an understanding of the current unmet needs, potential opportunity gains and resources for FLS in the context of other major long-term conditions in coastal localities.

The strength of this analysis is the use of routinely collected data that reflects real-world secondary fracture prevention based on 68 actively participating FLSs of various settings and levels of performance.

A major limitation is the aggregation of FLS performance within coastal and non-coastal settings, given there is significant variability of performance between FLSs. Our findings are dependent on the active participation of FLSs in the audit. Hence, FLSs may deliver effective secondary fracture prevention but not participate in the audit and so be classified as ‘no FLS’. However, given that 68 FLSs are actively participating, this is unlikely to be a significant misclassification bias. A second limitation of this study is the definition of a coastal FLS. An FLS was classified as coastal if it included one or more coastal towns. Some coastal FLSs also cover non-coastal towns but as the data is at FLS-level we have not been able to separate at town level which would have given more specific results. Finally, the dataset covers fractures in 2022, which precedes the NHS recommendations for abaloparatide [24] and the romosozumab-required NHS implementation in the latter quarter of 2022 [11].

In summary, these data support the inclusion of secondary fracture prevention as a coastal health priority in the NHS to deliver resourcing of FLSs as well as ensuring FLSs have the capability and capacity to personalise treatment recommendations in line with current NICE recommendations.
